# Nitric oxide and asymmetric dimethyl arginine (ADMA) levels in an experimental hydronephrotic kidney caused by unilateral partial ureteral obstruction

**DOI:** 10.1590/S1677-5538.IBJU.2015.0030

**Published:** 2016

**Authors:** Cabir Alan, Hasan Anil Kurt, Naci Topaloğlu, Ahmet Reşit Ersay, Dilek Ülker Çakir, Gökhan Baştürk

**Affiliations:** 1 Department of Urology, Medical Faculty, Canakkale Onsekiz Mart University, Turkey

**Keywords:** Ureteral Obstruction, Nitric Oxide, Rats, Superoxide Dismutase

## Abstract

**Aim:**

Our aim is to measure asymmetric dimethyl arginine and nitric oxide levels in rats with induced unilateral acute ureteral obstruction to research the effects on the kidney.

**Material and Methods:**

The study included 21 adolescent (average age 6 weeks) Sprague-Dawley male rats weighing between 240-290g divided at random into 3 groups. Group-1: Control group (n=6): underwent no procedures. Group-2: Sham group (n=6): underwent the same procedures as the experimental group without ureter and psoas muscle dissection. Group-3: Group with induced partial unilateral ureteral obstruction (n=9). All rats were sacrificed after 12 weeks. Superoxide dismutase enzyme activity and nitrite and nitrate salt levels were measured in renal tissue. Plasma nitrite-nitrate and ADMA levels were examined.

**Results:**

In the experimental group histopathological changes observed included renal pelvis dilatation, flattened papillae, sclerotic glomerulus and fibrosis. In the experimental group tissue SOD and blood ADMA levels were higher than the control and sham groups (p<0.05) while tissue NO and plasma NO values were lower than in the sham and control groups (p<0.05).

**Conclusion:**

Oxidative stress and disruption of NO synthesis play an important role in renal function and histopathological changes after obstructive renal disease. To prevent renal complications developing after obstructive nephropathy we believe that a new strategy may be research on reducing ADMA.

## INTRODUCTION

Ureteral obstructions are frequently observed in urology practice and without early diagnosis and treatment it may cause some serious complications (1-4). Many experimental studies have shown that retrograde glomerular reflux developing due to ureteral obstruction disrupts NO synthesis and in parallel affects renal function (5-8). However, how and by which mechanism NO synthesis is disrupted is still not fully known.

Asymmetric dimethyl arginine (ADMA) is a nitric oxide synthesis (NOS) inhibitor (9). The function of NOS in the body is provided by NO synthesis from L-arginine. In this reaction, occurring in the vascular endothelium, ADMA inhibits NOS activity by preventing L-arginine uptake into cells. In other words ADMA regulates the rate of NO formation (9). Impaired endothelium vasodilation, increased aggregation of platelets and increased monocyte adhesion provide endothelial dysfunction that increase ADMA (10). During ureteral obstruction, inflammatory mediators in renal parenchyma have been shown to increase due to retrograde glomerular reflux. This situation increases ADMA synthesis causing NO synthesis disruption, which causes an effect on renal function. In one study, 221 chronic renal failure patient’s serum ADMA levels were shown to be increased (11).

The aim of the study was to induce unilateral acute ureteral obstruction in rat models to measure ADMA and NO levels to research the effect on the kidney.

## MATERIAL AND METHODS

### Experimental Animals and Study Groups

This study began after permission was granted by Canakkale Onsekiz Mart University Animal Experiments Ethics Committee. The study used 21 adolescent (average 6 weeks) Sprague-Dawley male rats weighing from 240-290g. The animals were kept in standard laboratory conditions with stable temperature (18-21ºC) and humidity, 12 hours of light and 12 hours of darkness, with 3-4 rats in each cage fed with rat food and tap water.

The rats were randomly divided into 3 groups. Group-1: Control group (n=6): underwent no procedures. Group-2: Sham group (n=6): underwent the same procedures as the experimental group (the ureter and psoas muscle were palpated and left on their own anatomical position). Group-3: Experimental group: induced partial unilateral ureteral obstruction (PUUO) (n=9).

### Surgical Procedure

Rats used for partial unilateral ureteral obstruction (PUUO) and rats undergoing the sham operation were starved for 6 hours before the operation and only allowed water. The rats, in accordance with antiseptic rules, were operated on under laboratory conditions maintaining body temperature. All rats were anesthetized with intramuscular 50mg/kg ketamine hydrochloride (Ketalar, Eczacibasi) and then a 2cm area was shaved on bilateral abdominal wall and 10% Povidone iodine was used to clean the field.

**Figure 1:**
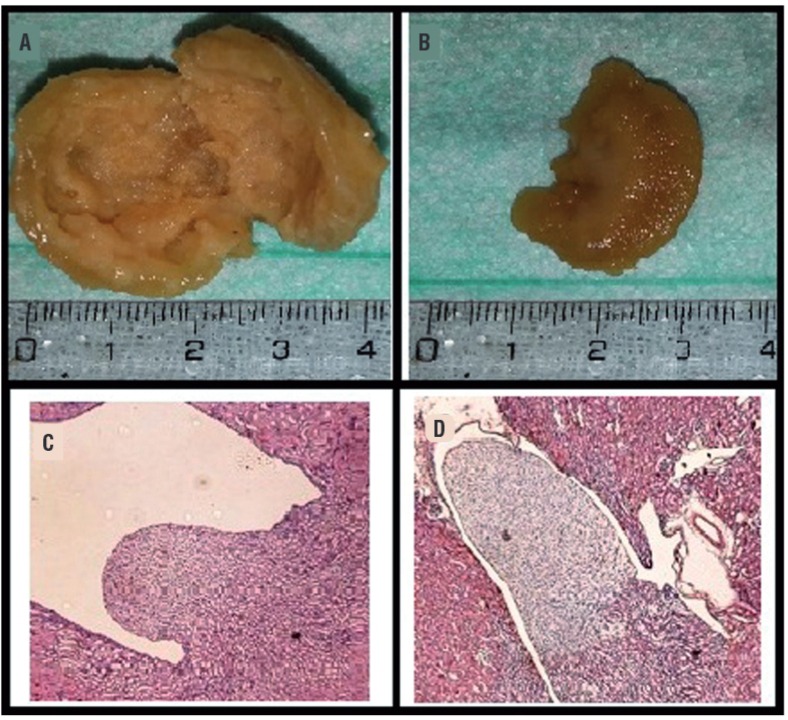
a) Macroscopic view of kidney tissue of experimental group; b) Macroscopic view of kidney tissue of control group; c) Hematoxilin and eosin (H&E) stained histopathological images of kidney tissue of experimental group; renal pelvis dilatation and flattening; d) Hematoxilin and eosin stained histopathological images of kidney tissue of control group; there was no pathological findings on renal papilla, cortex and medulla.

Partial unilateral ureteral obstruction (PUUO) model: A 2cm incision was performed at the anterior abdomen region. Skin above the linea alba, subdermis, abdominal anterior wall and peritoneum was incised to reach abdominal cavity and the left kidney was located. Fat and connective tissues were dissected to reveal the left ureter and psoas muscle. For PUUO rats, similar to the technique of Ulm and Miller (12), the psoas muscle under the left ureter was longitudinally dissected to form a groove (~15mm) and a small part of the left ureter was placed into the groove. Later the edges of the psoas muscle were fixed above the ureter with 5/0 silk suture. Thus the ureter was enclosed in a tunnel (Figure-1). In the sham model rats after the left ureter and psoas muscle were located, the procedure was completed without ureter and psoas muscle dissection. Later 3/0 catgut and 3/0 silk suture were used to close the abdomen in 2 layers. All rats were sacrificed after 12 weeks.

### Biochemical Tests

The kidney was dissected and excised and then stored at-80ºC. To test for biochemical changes, the levels of superoxide dismutase (SOD) enzyme activity and nitrite and nitrate salts which are end-products of NO were measured. To investigate the nitrite-nitrate and ADMA levels in plasma, 5cc blood samples from the ventricle of rats were centrifuged for 10 minutes at 3000rpm and the separated samples were stored at-40ºC until analysis.

### Tissue Homogenization

After tissue samples were weighed, for assay of total nitrite/nitrate and SOD samples, they were homogenized in 0.9% NaCl and 10% homogenates were prepared. For assay of total nitrite/nitrate and SOD, the prepared homogenates were centrifuged for 15 minutes at 15000rpm. Both assays were completed using samples of the supernatant liquid.

### Nitrite/nitrate

For measurement a “nitric oxide colorimetric assay” kit (Boehringer Mannheim) was used to evaluate at 540nm.

### Tissue SOD Activity Assay

Tissue SOD activity was measured at 560nm by modifying the method determined by Sun et al. (13).

## ADMA

ADMA levels in serum were measured by using a kit from BioVendor Research and Diagnostic Products (Cat. No: REA 201/96) manufactured by DLD Diagnostica GMBH (Germany). Results were determined by the ELISA method and reported as nanogram per millilitre (ng/mL).

### Histopathological Investigation

After tissue samples were cleaned with saline they were immediately fixed in 10% formaldehyde solution at room temperature for 72 hours and prepared for routine light microscope examination. The tissue samples were dehydrated with alcohol and cleaned with xylene before being immersed in paraffin.

Five-micrometer slices of the tissues were made. The tissues were stained with hematoxyline-eosine (H&E) and periodic acid-Schiff (PAS) and the sections were examined and photographed with a light microscope.

### Statistical Evaluation

The Statistical Package for the Social Sciences 20.0 for Windows (SPSS Inc., Chicago, IL, USA) was used for statistical analysis. All values were given as mean±standard deviation. Kruskal-Wallis test was used for variance analyses. Mann-Whitney U test was used for dual comparisons between groups. Statistical significance was accepted as p<0.05.

## RESULTS

While there was no pathological changes in the kidney sections from the control group and sham group, in the experimental group induced PUUO, on histopathological examination of the H&E dyed kidney fibrosis, inflammation, flattening of the renal papillae and dilatation of the renal pelvis were observed with varying degrees On kidney sections stained with PAS sclerotic glomerular changes, inflammation and fibrotic changes were observed (Figure-1).

**Figure 2:**
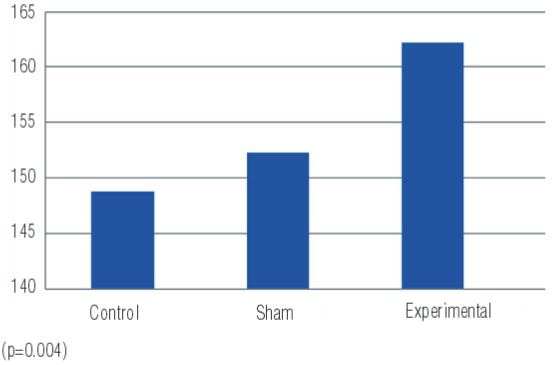
SOD values measured in tissue samples of the groups (μmoL/g tissue).

Linked to increased oxidative stress the tissue SOD values, the enzyme with primary antioxidant properties, are summarized in Table-1. In the experimental group with induced PUUO, tissue levels of SOD were higher than in the sham and control groups (p=0.001) (Figure-2). Comparing the groups, there were differences between the experimental and control groups (p=0.001) and between the experimental and sham groups (p=0.003) (Table-1).

**Table-1:** Biochemical results of the three groups.

	Control		Sham		Experimental		
	
	median	ss(±)	median	ss(±)	median	ss(±)	p
**Tissue nitrate**	0.45	0.04	0.33	0.06	0.28	0.02	0.0003
**Plasma nitrate**	31.72	2.36	21.28	3.16	16.86	0.57	0.0003
**SOD**	148.82	3.5	152.28	3.9	162.27	4.5	0.001
**ADMA**	0.82	0.14	0.92	0.82	2.7	0.59	0.001

**Figure 3:**
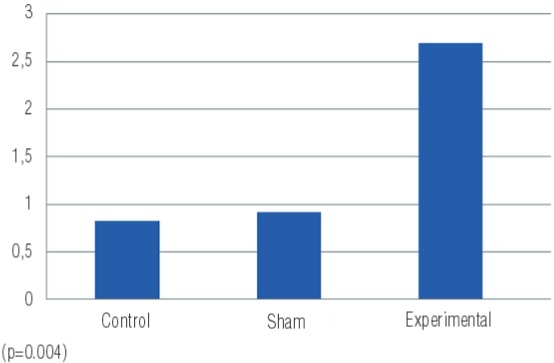
ADMA values measured in blood samples of the groups (ng/mL).

There was a clear increase in ADMA values measured in blood in the experimental group compared to the control and sham groups (p=0.001) (Figure-3). In the sham group there were higher levels of ADMA than in the control group (Table-1).

**Figure 4:**
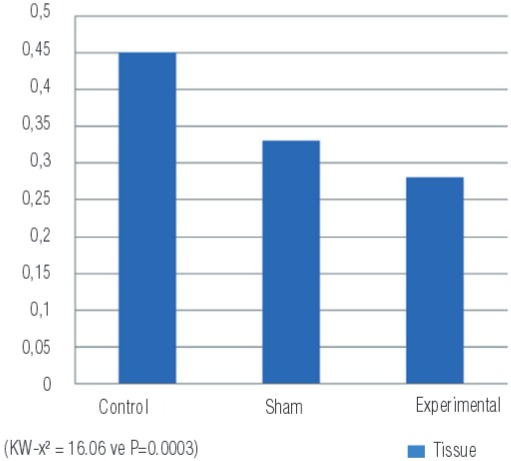
Nitrite/nitrate levels measured in tissue samples of the groups (µmoL/g tissue).

Nitrite/nitrate levels, end products of NO, were identified in both renal tissue and in plasma. The nitrite/nitrate values in tissue were lower in the PUUO induced experimental group compared to the sham and control groups (p=0.0003) (Figure-4) (Table-1).

**Figure 5:**
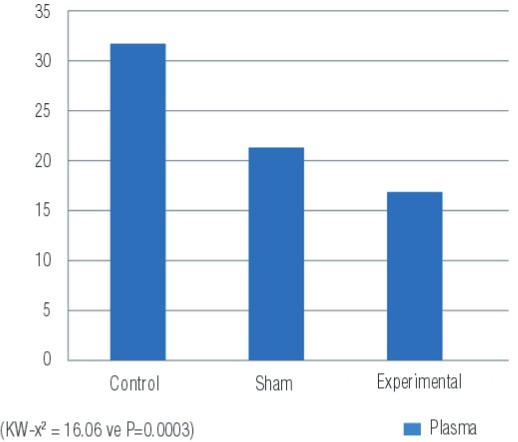
Nitrite/nitrate levels measured in blood samples of the groups (µmol/L).

Assay results of plasma nitrite/nitrate levels are shown in Figure-5. These results show the lowest plasma levels were found in the experimental group, followed by the sham group and the highest levels were in the control group. There was a significant difference between the groups (p=0.0003) (Table-1).

## DISCUSSION

Different etiologic factors such as urinary tract stones, ureteropelvic junction or ureterovesical junction stenosis, tumors and iatrogenic factors lead to upper uriner tract obstruction that can cause organ failure in urology practice. Treatment planning is depend on duration, degree and level of obstruction. Upper urinary tract patologies have a broad treatment spectrum. The primary aim of treatment is to protect or recover the functional reserve of kidneys by relieving the obstruction. In case of prolonged obstruction, functional reserve of kidney can decrease so much that nephrectomy is required to prevent morbidities of dysfunctional kidney.

Fibrosis, collagen and extracellular matrix components accumulation are the most leading interstitial changes in upper urinary tract obsruction. Besides cellular composition changes of interstitium, many biologically active molecules changes occur with interstitial fibrosis. It is considered that obliteration of tubules and interstitial capillaries due to interstitial fibrosis are the major determinants of renal function failure in kidney disease. Claesson et al. (14) induced left chronic partial ureteral obstruction in newborn rats. After two weeks they began to observe histopathological changes from ureteral obstruction and they reported that primarily papilla deformation occurred. In our study the rats with induced PUUO were sacrificed after 12 weeks. In the hydronephrotic kidney, histopathological changes observed included renal pelvis dilatation, flattened papillae, sclerotic glomerulus and fibrosis.

In upper urinary tract obstructions, increased ureteral pressure is the first stage of damage that leads to renal blood flow reduction resulting in tissue ischemia, cellular atrophy and eventually necrosis. TNF-α is a potent proinflammatory cytokine that is capable of stimulating renal tubular cell apoptosis and infiltration of inflammatory cells during ischemic renal injury. After the development of obstructive nephropathy, it is reported that there is a role of factors such as prostaglandins (PG), angiotensin (ANG) II, growth factors and NO in the accumulation of free oxygen radicals and leukocyte infiltration (15). ROS may release vasoconstrictor bioactive lipids such as prostaglandin, thromboxane and platelet activating factors and inactivate NO inducing a reduction in glomerular blood flow and glomerular infiltration rate (16, 17). Ricardo et al. (18) after inducing a unilateral ureteral obstruction model in rats reported that ROS and overproduction of tubular irregular antioxidant enzymes cause increased intrarenal oxidative stress, leading to fibrogenesis, over expression of fibrogenic cytokines and loss of tubular shape. Kinter et al. (19) in a UUO model found high levels of antioxidant enzymes (catalase, SOD, glutathione peroxidase) which can protect against the harmful effects of ROS. After UUO, ROS production was greater than the protective capacity of the antioxidant enzymes increasing renal damage with tubular atrophy and interstitial fibrosis observed. In our study a marker of oxidative stress (SOD level in tissue) was higher in the experimental group compared to the sham and control groups (p=0.001 and p=0.003). It was shown our study that upper urinary tract obstruction led to oxidative stress with disturbance of NO synthesis, creating fibrosis which could lead to renal damage.

Asymmetric dimethylarginine (ADMA) is an amino acid, like arginine, found in plasma, urine and tissue. In 1992, Vallance et al. (4) identified ADMA in human plasma and urine endothelial tissue as endogenous inhibitors of endothelial nitric oxide synthase (eNOS). It was the first time that the importance of ADMA as endogenous inhibitör of NOS in patient with end stage renal failure was cited by Vallance et al. (1). In these patients, increased plasma ADMA levels were reduced by dialysis with improvement of endothelial function. Later studies showed many times the relationship between increased ADMA levels and endothelial vasodilator dysfunction. In the endothelium NO is produced from L-arginine via the endothelial isoform (eNOS) of nitric oxide synthase. In humans NO synthesis may be disrupted by asymmetric dimethylarginine (ADMA), removing the endogenically-formed compound L-arginine from substrate linking points inhibiting NOS activity. An infusion of ADMA disrupts vasodilation in the endothelium. Increased ADMA levels in plasma are not only linked to endothelial dysfunction, but also related to increased oxidative stress (20). Boger et al. (21) found that oxidative stress damaged the cystein aminoacid in the active region of the DDAH enzyme responsible for ADMA catabolism reducing enzyme activity and thus reducing the ADMA disintegration. As a result of increasing oxidative stress in many degenerative diseases, ADMA levels were found to be high. In ureter obstruction, free oxygen radicals increase, as shown by many studies mentioned above (18, 19, 22). In our study, the findings are in accordance with these studies. Plasma ADMA levels in the experimental group were higher than in the sham and control groups (p=0.001 and p=0.001, respectively). ADMA inhibits NOS activity causing a reduction in NO levels, and as a result, disruption of endothelial function. Lin et al. (10) showed increased ADMA concentration in endothelial dysfunction was related to increased ROS production in plasma. In addition Takiuchi et al. (23) showed that endothelial dysfunction in coronary and peripheral vein diseases was linked to increased ADMA levels in plasma. The findings of our study are consistent with all these studies. When compared the experimental group to control and sham groups, we found increased both ADMA and SOD levels (p<0.05).

Nitric oxide is used in mitochondrial respiration, membrane transport and cellular energy stages, with a role in glomerular hemodynamic regulation, tubular transport and tubule-glomerular reabsorption and as a result is reported to play an important role in kidney physiology and pathology (24). Cherla and Jaimes (25) showed that when given the NO percursor, arginine, the synthesized NO had a protective effect against kidney damage. The reduction in the amount of NO in the kidney was an important effect on the progression of renal damage, and they reported that NOS inhibition led to hypertension and sodium retention (24). Many studies have examined the role of NO in the hemodynamic response to UUO. Lanzone et al. (6) found L-NMMA administration before UUO reduced the first increase in renal blood flow. When L-NMMA infusion was discontinued the renal blood flow increase resolved within 10 minutes. These findings prove the role of NO in pre-glomerular vascular resistance reduction after ureteral occlusion. Another study on NO created by eNOS in the kidney found that it played a basic role in protecting against renal fibrosis developing as a response to UUO (7). The reduction in biological effectiveness of NO increases oxidative stress (8). In our study the products of NO, nitrite and nitrate, were examined in tissue and plasma. The nitrite/nitrate levels in tissue and plasma were lower in the experimental group compared to the control and sham groups (p=0.0003 and p=0.0003). This situation is an indirect indicator of decreased NO levels in upper urinary tract obstruction model. According to our findings, reduction of NO depended on both increase in ADMA levels and oxidative stress.

## CONCLUSIONS

Oxidative stress and disruption of NO synthesis play an important role in renal function and histopathological changes after obstructive uropathy. ADMA is an endogenous inhibitor of NOS. Increased ADMA levels show the inhibitor effect on NOS, causing a fall in NO levels in the kidney. We believe that methods to reduce ADMA may be a new strategy to prevent renal complications that develop after obstructive nephropathy.

## References

[1] Chevalier RL, Forbes MS, Thornhill BA (2009). Ureteral obstruction as a model of renal interstitial fibrosis and obstructive nephropathy. Kidney Int.

[2] Wongmekiat O, Leelarungrayub D, Thamprasert K (2013). Alpha-lipoic acid attenuates renal injury in rats with obstructive nephropathy. Biomed Res Int.

[3] Coplen DE, Synder HM, Ashcraft, Murphy, Sharp, Sigalet, Synder (2000). Ureteral Obstruction and Malformations. Pediatric Surgery.

[4] Chevalier RL, Thornhill BA, Gomez RA (1992). EDRF modulates renal hemodynamics during unilateral ureteral obstruction in the rat. Kidney Int.

[5] Klahr S (2000). Obstructive nephropathy. Intern Med.

[6] Lanzone JA, Gulmi FA, Chou SY, Mooppan UM, Kim H (1995). Renal hemodynamics in acute unilateral ureteral obstruction: contribution of endothelium-derived relaxing factor. J Urol.

[7] Ekinci S, Ciftci AO, Atilla P, Muftuoglu S, Senocak ME, Buyukpamukcu N (2003). Ureteropelvic junction obstruction causes histologic alterations in contralateral kidney. J Pediatr Surg.

[8] Araujo M, Welch WJ (2006). Oxidative stress and nitric oxide in kidney function. Curr Opin Nephrol Hypertens.

[9] Vallance P, Leiper J (2004). Cardiovascular biology of the asymmetric dimethylarginine:dimethylarginine dimethylaminohydrolase pathway. Arterioscler Thromb Vasc Biol.

[10] Lin KY, Ito A, Asagami T, Tsao PS, Adimoolam S, Kimoto M (2002). Impaired nitric oxide synthase pathway in diabetes mellitus: role of asymmetric dimethylarginine and dimethylarginine dimethylaminohydrolase. Circulation.

[11] Fleck C, Schweitzer F, Karge E, Busch M, Stein G (2003). Serum concentrations of asymmetric (ADMA) and symmetric (SDMA) dimethylarginine in patients with chronic kidney diseases. Clin Chim Acta.

[12] Ulm AH, Miller F (1962). An operation to produce experimental reversible hydronephrosis in dogs. J Urol.

[13] Sun Y, Oberley LW, Li Y (1988). A simple method for clinical assay of superoxide dismutase. Clin Chem.

[14] Chertin B, Rolle U, Farkas A, Puri P (2002). The role of nitric oxide in reflux nephropathy. Pediatr Surg Int.

[15] Chevalier RL (1999). Molecular and cellular pathophysiology of obstructive nephropathy. Pediatr Nephrol.

[16] Baud L, Ardaillou R (1993). Involvement of reactive oxygen species in kidney damage. Br Med Bull.

[17] Rabl H, Khoschsorur G, Colombo T, Petritsch P, Rauchenwald M, Költringer P (1993). A multivitamin infusion prevents lipid peroxidation and improves transplantation performance. Kidney Int.

[18] Ricardo SD, Diamond JR (1998). The role of macrophages and reactive oxygen species in experimental hydronephrosis. Semin Nephrol.

[19] Kinter M, Wolstenholme JT, Thornhill BA, Newton EA, McCormick ML, Chevalier RL (1999). Unilateral ureteral obstruction impairs renal antioxidant enzyme activation during sodium depletion. Kidney Int.

[20] Böger RH, Schwedhelm E, Maas R, Quispe-Bravo S, Skamira C (2005). ADMA and oxidative stress may relate to the progression of renal disease: rationale and design of the VIVALDI study. Vasc Med.

[21] Böger RH, Maas R, Schulze F, Schwedhelm E (2005). Elevated levels of asymmetric dimethylarginine (ADMA) as a marker of cardiovascular disease and mortality. Clin Chem Lab Med.

[22] Kawada N, Moriyama T, Ando A, Fukunaga M, Miyata T, Kurokawa K (1999). Increased oxidative stress in mouse kidneys with unilateral ureteral obstruction. Kidney Int.

[23] Takiuchi S, Fujii H, Kamide K, Horio T, Nakatani S, Hiuge A (2004). Plasma asymmetric dimethylarginine and coronary and peripheral endothelial dysfunction in hypertensive patients. Am J Hypertens.

[24] Klahr S (2001). The role of nitric oxide in hypertension and renal disease progression. Nephrol Dial Transplant.

[25] Cherla G, Jaimes EA (2004). Role of L-arginine in the pathogenesis and treatment of renal disease. J Nutr.

